# Comparative study of the outcomes of robotic versus laparoscopic distal gastrectomy with hand-sewn anastomosis in Billroth-I reconstruction

**DOI:** 10.1186/s12893-025-03373-y

**Published:** 2025-11-25

**Authors:** Zhenxing Zhang, Dunbo Liu, Shan Wang, Bo Wang, Zhiwei Sun, Yue Meng, Yang Song, Guohang Dai, Dongming Liu, Qianshi Zhang, Shuangyi Ren

**Affiliations:** 1 Department of Gastrointestinal Surgery, The Second Hospital of Dalian Medical University, Dalian, 116023 China; 2 Department of Gastrointestinal Surgery, Shaoxing People’s Hospital, Shaoxing, 312000 China; 3 Department of Gastrointestinal Surgery, Linyi Central Hospital, Linyi, 276400 China; 4 Department of Special Examination, Shaoxing People’s Hospital, Shaoxing, 312000 China; 5 Department of General Surgery, The Second Affiliated Hospital of Dalian Medical University, 467 Zhongshan Road, Dalian, 116023 China

**Keywords:** Laparoscopic gastrectomy, Robotic gastrectomy, D2 lymphadenectomy, Billroth-I, Gastric cancer

## Abstract

**Purpose:**

Total laparoscopic distal gastrectomy hand-sewn anastomosis (LDG-HA) for gastric cancer (GC) is safe and effective. The objectives of our study were to investigate the efficacy and safety of laparoscopic versus robotic distal gastrectomy hand-sewn anastomosis (RDG-HA) in Billroth-I reconstruction.

**Methods:**

We retrospectively analyzed the clinical data of 95 patients treated with LDG-HA (*n* = 55) and RDG-HA (*n* = 40) for GC from 09/2018 to 06/2021. The effects on baseline, pathology, perioperative data, short-term outcomes, long-term outcomes and 5-year oncologic outcomes follow-up were analyzed.

**Results:**

The difference between the clinical-pathological characteristics of the two groups was not statistically significant (*P* > 0.05). The RDG-HA group was associated with a shorter anastomosis time (20.80 min vs. 23.36 min, *P* = 0.001) but a longer operative time (176.55 vs. 151.86 min, *P* = 0.003) and a higher cost (97661.66 CNY vs. 71082.63 CNY, *P* = 0.000). The differences of complications, 5-year follow-up overall survival and disease-free survival rates between the two groups were not statistically significant.

**Conclusion:**

RDG-HA is safe and effective with a faster procedure of reconstruction but much more expensive.

## Introduction

Cancer remains the number two killer worldwide after cardiovascular disease [1]. Worldwide, the mortality rate of GC has shown a clear downward trend, but in China, the overall incidence of GC ranks fourth among all tumors and the mortality rate ranks third [2]. Currently, the treatment of GC is still based on surgery as the main comprehensive treatment. The CLASS-01 study in China, a multicenter study demonstrating equivalent safety and efficacy of laparoscopic versus open surgery for radical treatment of distal GC [3]. Since the first case of delta-shaped Billroth-I anastomosis in totally laparoscopic distal gastrectomy (LDG) was reported by Kanaya in 2002 [4], and the fact that Billroth-I reconstruction after gastrectomy is more physiologic, more and more surgeons were keen to take up totally laparoscopic Billroth-I reconstruction. A randomized controlled study from China suggests that total LDG for digestive tract reconstruction with Delta-shaped Billroth-I anastomosis is simple and easy to perform and offers advantages in postoperative recovery of gastrointestinal function [5]. In addition to Delta-shaped anastomosis, complete hand-sewn anastomosis (HA) is also a modality of Billroth-I. In 2003, Takiguchi reported the first totally laparoscopic Billroth-I HA and Hashizume reported the first totally robotic HA [6, 7]. Studies on Billroth-I HA after that time are scarce, with only a few case reports. The robotic surgical system is a new platform for minimally invasive surgery. Among them, the Da Vinci Surgical System is the most widely used robotic surgical system in clinical practice due to its ability to provide high-definition three-dimensional (3D) imaging of the joint motion and eliminating physiologic tremor [8]. Zhang et al. found that the application of LDG-HA was a safe and feasible method because it not only ensured the completion of Billroth-I anastomosis, but also reduced the cost of the surgery [9]. Here we firstly conducted this study on the short- and long-term outcomes of robotic distal gastrectomy (RDG) compared with LDG with HA.

## Patients and methods

### Patients and study design

**Fig. 1:**
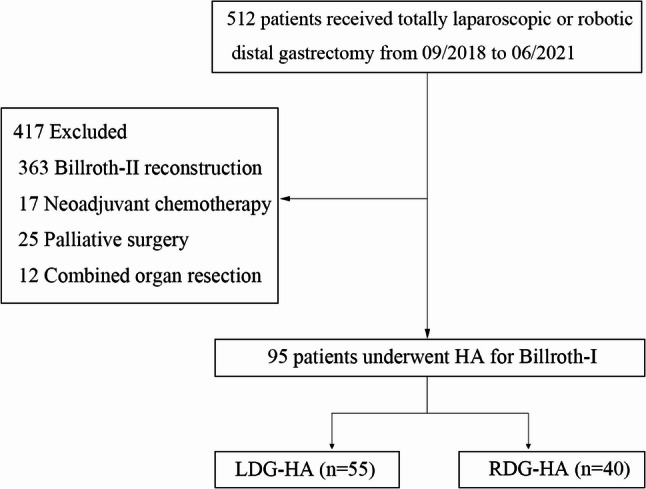
The flow diagram of the study patient selection process. A total of 512 patients underwent totally laparoscopic or robotic distal gastrectomy from 09/2018 to 06/2021 were retrospectively collected. 417 patients were excluded, including 363 Billroth-II reconstructions, 17 neoadjuvant chemotherapy, 25 palliative surgery, and 12 combined organ resections. Finally, a total of 95 patients underwent HA for Billroth-I were divided into the LDG-HA group (*n* = 55) and RDG-HA group (*n* = 40)

This retrospective cohort study included 95 patients, each patient diagnosed with distal GC (tumor located in the lower part of the stomach without invasion of the pylorus) had signed an informed consent form and underwent Billroth-I reconstruction with LDG-HA or RDG-HA in The Second Hospital of Dalian Medical University from 09/2018 to 06/2021 (the patients selection process was detailed in Fig. 1). All surgeries were performed by a surgeon with experience in more than 5000 laparoscopic and 1000 robotic surgeries. The clinical-pathological characteristics (age, gender, body mass index (BMI), hemoglobin (Hb), white blood cells (WBC), albumin (ALB), diameter of tumor, previous abdominal surgery, obstruction, bleeding, hypertension, diabetes, smoking, alcohol consumption, American Society of Anesthesiologists (ASA) score, and postoperative pathological staging), perioperative data (operative time, anastomosis time, operation blood loss, diameter, number of lymph nodes, time to first flatus, time to liquid diet, postoperative hospital days, and inpatient costs), short-term complications (abdominal infection, intra-abdominal hemorrhage, anastomotic leakage, anastomotic hemorrhage, gastroparesis, diarrhea and pneumonia), long-term complication (gastric retention, reflux and anastomotic stenosis), and 5-year oncologic outcomes follow-up including Disease-free survival (DFS) and overall survival (OS) were retrospectively analyzed.

## Methods

**Fig. 2:**
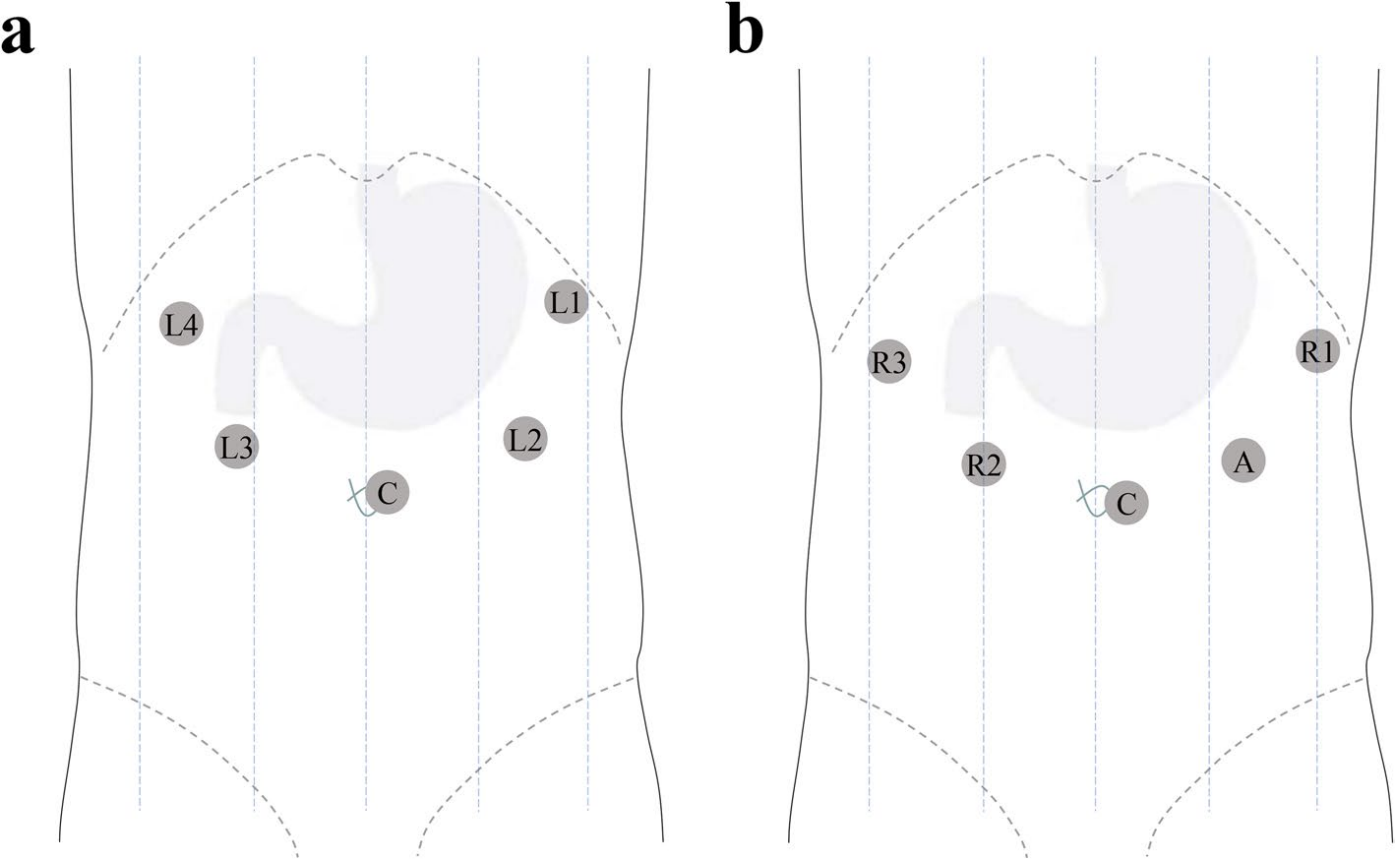
Trocar placement. **a** Trocar placement for LDG-HA L1/L2, operator’s right/left hand; L3/L4, assistant’s right/left hand; C, camera. **b** Trocar placement for RDG-HA. R1, arm 1; R2, arm 2; R3, arm 3; A, assistant port; C, camera

RDG-HA was performed using Da Vinci ^®^ Si Surgical Systems, while LDG-HA using a 3D laparoscopic system (AESCULAP) with 3D glasses worn by every surgeon and nurse. Anesthesia was achieved by endotracheal intubation and intravenous drugs, and the patient was then placed in the supine position. The five-port technique was used in both LDG-HA and RDG-HA (Fig. 2a, b). Pneumoperitoneal pressure was maintained at 12 mmHg, and D2 lymphadenectomy for distal gastrectomy with hand-sewn for Billroth-I was performed in all patients. The procedures were all carefully performed with the intention to treat in accordance with Declaration of Helsinki. The medical team acknowledge the robot surgery and the informed consents for surgery were agreed by all patients. The study was approved by the Academic Ethics Committee of the Second Hospital of Dalian Medical University.

### LDG-HA details

After completion of lymph node dissection, the specimen was prepared for GI reconstruction. It should be emphasized that preliminary docking of the gastric remnant to the duodenal stump was required to check anastomotic tension and hematology prior to Billroth-I reconstruction. In the first step, the posterior wall of the anastomosis was closed by continuous plasma-muscle layer sutures using single-needle absorbable barbed sutures. In the second step, the posterior wall of the anastomosis was closed with a continuous full-layer suture using another single-needle absorbable barbed suture. In the third step, the same single-needle absorbable barbed suture was performed to close the anterior wall of the anastomosis with continuous full-layer sutures. In the final step, the stump of the lesser curvature of the stomach along with the anterior wall of the anastomosis was sutured with the first single-needle absorbable barbed suture in a continuous plasma-muscle layer.

### RDG-HA details

**Fig. 3:**
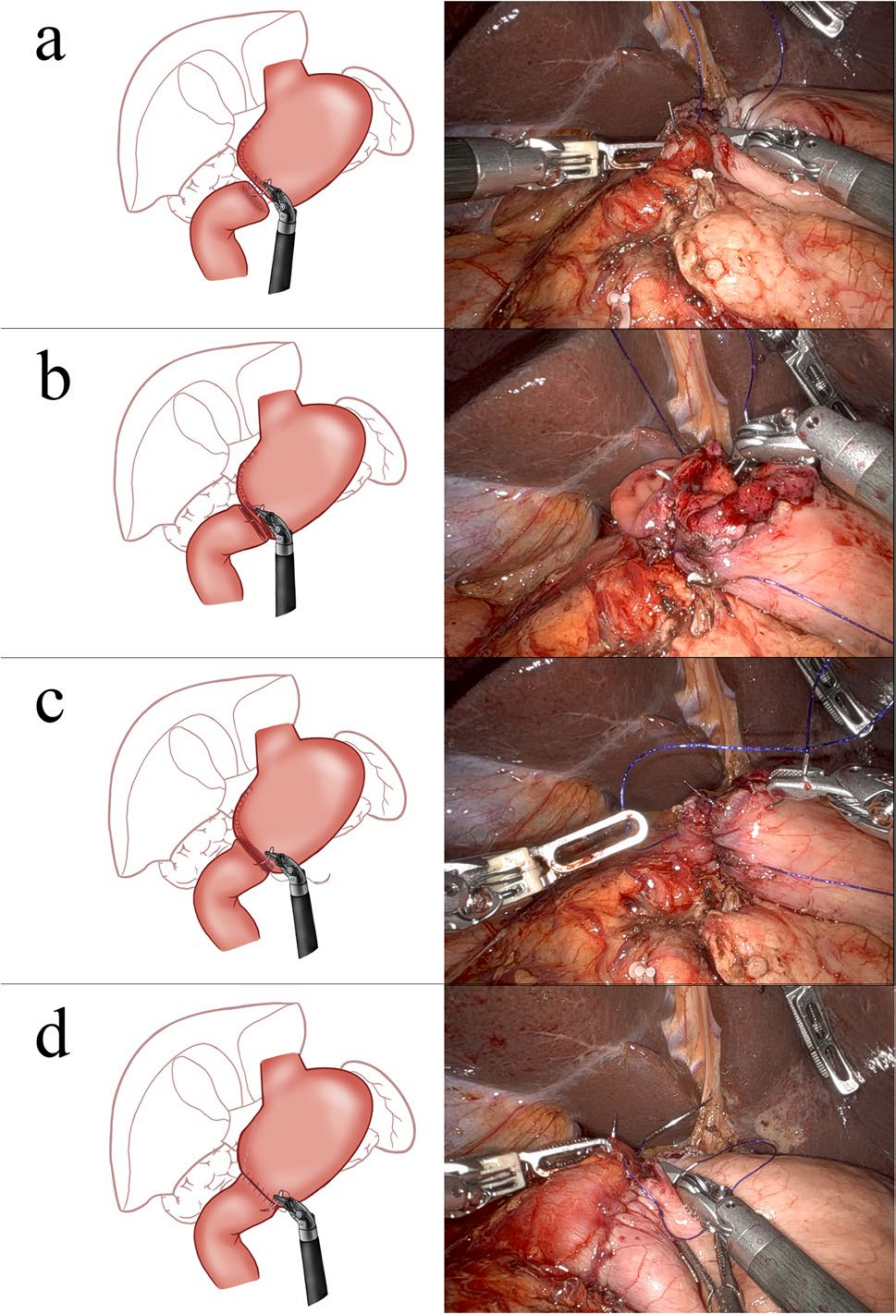
The detailed process of RDG-HA. **a** The posterior wall of the anastomosis was closed by continuous plasma-muscle layer sutures using single-needle absorbable barbed sutures. **b** The posterior wall of the anastomosis was closed with a continuous full-layer suture. **c** The same barbed suture was performed to close the anterior wall of the anastomosis with continuous full-layer sutures. **d** The stump of the lesser curvature along with the anterior wall of the anastomosis was sutured with the first single-needle absorbable barbed suture in a continuous plasma-muscle layer

The Billroth-I hand-sewn anastomosis under the robot was performed in the same manner as the reinforcement of the gastric lesser curvature stump. The detailed process and diagrams were shown in Fig. 3.

### Definitions

The operative time was measured from incision of the skin to closure of the wound and was consistent with the anesthesia transcript. The anastomosis time was measured from the time the specimen was loaded into the specimen bag to the time the reconstruction was completed and consistent with the surgical record. Standard D2 lymphadenectomy with dissection of the No. 1, 3, 4sb, 4 d, 5, 6, 7, 8a, 9, 11p, and 12a lymph nodes was performed according to the Japanese Classification of Gastric Carcinoma, 15th edition [10]. Postoperative pathology was staged according to the 8th American Joint Committee on Cancer staging [11]. Postoperative complications were graded according to the Clavien-Dindo classification [12]. DFS time was defined as the duration from surgery to tumor relapse or progression. OS time was defined as the duration from the operation to death of any cause.

Statistical analysis (Data analysis).

SPSS (SPSS Inc., version 26.0, Chicago, IL, USA) was used for all analyses. Continuous variables were expressed as mean ± standard deviation (mean ± SD), and comparisons between groups were made by t-test or Mann-Whitney test. Categorical data were expressed as n (%) and analyzed using the chi-square test or Fisher’s exact test. The OS and DFS were analyzed using the Kaplan-Meier method, and differences were analyzed using the log-rank test. Statistical significance was defined as *P* < 0.05.

## Results

### Clinical-pathologic characteristics

There were no statistical differences between the LDG-HA (*n* = 55) and RDG-HA (*n* = 40) groups in terms of clinical-pathological characteristics such as age, gender, BMI, Hb, ALB, tumor diameter, previous abdominal surgeries, obstruction, bleeding, hypertension, diabetes, smoking, alcohol consumption, ASA scores, postoperative pathological staging, degree of differentiation, vessel invasion and nerve invasion (Table 1).

**Table 1:** Clinical-pathological characteristics

			LDG-HA(*n* = 55)	RDG-HA(*n* = 40)	*P*
Age			64.309 ± 11.439	63.7 ± 9.455	0.777
Gender		Male	37(67.3)	33(82.5)	0.096
		Female	18(32.7)	7(17.5)	
BMI			23.479 ± 2.872	23.486 ± 2.615	0.989
Hemoglobin			131.6 ± 26.595	137.65 ± 21.265	0.222
Albumin			41.609 ± 4.35	42.052 ± 4.987	0.654
White blood cells			5.619 ± 1.408	5.566 ± 1.238	0.845
Previous abdominal surgery			10(18.2)	3(7.5)	0.135
Obstruction			1(1.8)	1(2.5)	0.819
Bleeding			2(3.6)	1(2.5)	0.755
Hypertension			14(25.5)	9(22.5)	0.740
Diabetes			8(14.5)	8(20.0)	0.483
Smoking			13(23.6)	10(25.0)	0.878
Alcohol consumption			11(20.0)	5(12.5)	0.335
ASA		I	1(1.8)	0(0.0)	0.682
		II	48(87.3)	36(90.0)	
		III	6(10.9)	4(10.0)	
Diameter of tumor			2.518 ± 1.44	2.87 ± 1.335	0.223
Pathological staging	pStage	I	35(63.6)	29(72.5)	0.159
		II	10(18.2)	9(22.5)	
		III	10(18.2)	2(5.0)	
	Diferentiation	High	4(7.3)	4(10.0)	0.903
		Moderate	22(40.0)	15(37.5)	
		Poor	24(43.6)	16(40.0)	
		Other	5(9.1)	5(12.5)	
		Vessel invasion	19(34.5)	14(35.0)	0.963
		Nerve invasion	12(21.8)	9(22.5)	0.937
	T Staging	T1	28(50.9)	20(50.0)	0.813
		T2	11(20.0)	11(27.5)	
		T3	9(16.4)	5(12.5)	
		T4	7(12.7)	4(10.0)	
	N Staging	N0	34(61.8)	29(72.5)	0.265
		N1	8(14.5)	6(15)	
		N2	5(9.1)	4(10.0)	
		N3	8(14.5)	1(2.5)	

### Surgical outcomes and postoperative rehabilitation

Perioperative data, including surgical outcomes and postoperative rehabilitation, for LDG-HA and RDG-HA are presented in Table 2. The operation time in the RDG-HA group was significantly slower than that in the LDG-HA group (176.55 ± 42.30 vs. 151.86 ± 31.12 min, *P* = 0.003). However, the time of reconstruction in the RDG-HA group was significantly faster than that in the LDG-HA group (20.80 ± 2.35 vs. 23.36 ± 4.73 min, *P* = 0.001). In addition, total hospitalization cost was significantly higher in the RDG-HA group than in the LDG-HA group (97661.66 ± 14826.09 vs. 71082.63 ± 18296.88 CNY, *P* = 0.000). There were no significant differences between the LDG-HA and RDG-HA groups in terms of intraoperative blood loss, number of lymph nodes, time to first flatus, time to liquid diet and time to semi-liquid diet.

**Table 2:** Surgical outcomes and postoperative rehabilitation

	LDG-HA(*n* = 55)	RDG-HA(*n* = 40)	*P*
Operation time (min)	151.86 ± 31.12(135–170)^a^	176.55 ± 42.30(150–180)^a^	0.003
Reconstruction time (min)	23.36 ± 4.73(20–25)^a^	20.80 ± 2.35(20–22)^a^	0.001
Intraoperative blood loss (ml)	71.27 ± 42.61	60.13 ± 41.27	0.186
Number of lymph nodes	32.35 ± 12.58	33.30 ± 10.72	0.692
Time to frst flatus (Days)	1.93 ± 0.63	1.95 ± 0.50	0.846
Time to liquid diet (Days)	1.51 ± 0.54	1.53 ± 0.51	0.883
Time to semiliquid diet (Days)	2.89 ± 0.57	2.98 ± 0.70	0.533
Postoperative hospital stay (Days)	10.18 ± 6.59	9.00 ± 4.09	0.285
Hospital cost (CNY)	71082.63 ± 18296.88(63462.88–69814.11.88.11)^a^	97661.66 ± 14826.09(88523.68–102699.1.68.1)^a^	0.000

^a^95% confidence interval

### Short-term postoperative complications

The postoperative short-term complication rates were 32.5% and 30.9% in the RDG-HA and LDG-HA groups, respectively. For short-term complications, we stratified the study according to the Clavien-Dindo classification, extra-gastric systemic complications (pneumonia and other systemic complications such as: incision infection, diarrhea, cardiovascular stroke, cystitis, hepatic insufficiency, and small-bowel obstruction), and surgical-related complications (abdominal infection/pancreatic leakage, intra-abdominal hemorrhage, anastomotic leakage, anastomotic hemorrhage, anastomotic edema and gastroparesis), and there was no statistically significant difference between the RDG-HA and LDG-HA groups (Table 3). All patients who developed short-term complications were successfully treated and discharged from the hospital after treatment.

**Table 3:** Short-term postoperative complications

			LDG-HA(*n* = 55)	RDG-HA(*n* = 40)	*P*
Short-term	Overall	Overall	17(30.9)	13(32.5)	0.896
		I	3(5.5)	3(7.5)	
		II	8(14.5)	7(17.5)	
		IIIA	5(9.1)	3(7.5)	
		IIIB	1(1.8)	0(0.0)	
		IV/V	0(0.0)	0(0.0)	
	Extra-gastric system	Pneumonia	3(5.5)	3(7.5)	0.686
		Other systems	10(18.2)	5(12.5)	0.453
	Surgery-related	Abdominal infection/pancreatic leakage	1(1.8)	1(2.5)	0.819
		Intra-abdominal hemorrhage	1(1.8)	0(0.0)	0.391
		Anastomotic leakage	3(5.5)	4(10.0)	0.402
		Anastomotic hemorrhage	1(1.8)	0(0.0)	0.391
		Anastomotic edema	2(3.6)	1(2.5)	0.755
		Gastroparesis	3 (5.5)	1(2.5)	0.479

### Long-term postoperative complications and oncologic outcomes

For long-term complications, all patients in both groups underwent gastroscopy 1 year after surgery. All patients were required to fast for 12 h before gastroscopy, and the presence of gastric retention, gastroesophageal reflux, and anastomotic stenosis was determined by observing the residual gastric fluid, bile residue, and anastomotic size. There was no statistically significant difference in the long-term complications in either stratified analysis or overall incidence (23.5% vs. 25.5%, *P* = 0.837) in the RDG-HA and LDG-HA groups (Table 4).

**Table 4:** Long-term postoperative complications

		LDG-HA(*n* = 47)	RDG-HA(*n* = 34)	*P*
Long-term	Overall	12(25.5)	8(23.5)	0.837
	Gastric retention	10(21.3)	5(14.7)	0.452
	Reflux	3(6.4)	5(14.7)	0.215
	Anastomotic stenosis	0	0	NA

**Fig. 4:**
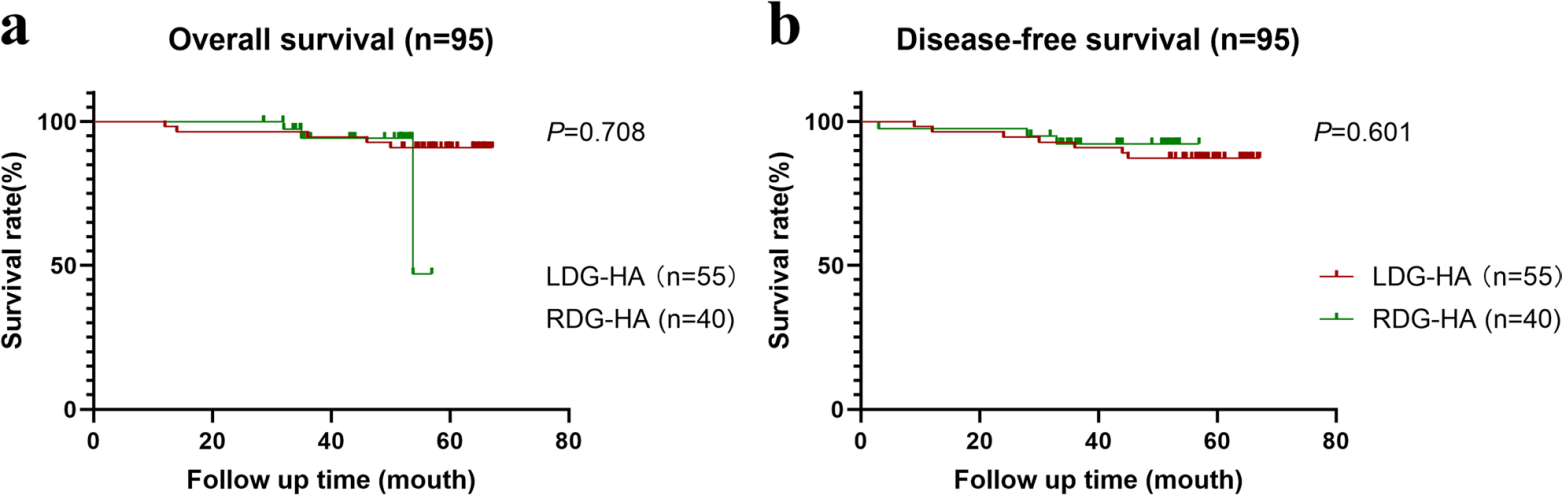
5-year postoperative follow-up. **a** showed the overall survival and **b** showed the disease-free survival. A total of 81 cases were followed up, with 47 cases in the LDG-HA group in red and 34 cases in the RDG-HA group in green

We conducted a 5-year postoperative follow-up to investigate OS and PFS in 95 patients, 55 in the LDG-HA group and 40 in the RDG-HA group. The median follow-up time was 54.4 months (12–67 months). There was no statistically significant difference in OS (*P* = 0.708) and DFS (*P* = 0.601) survival rates (Fig. 4).

## Discussion

In 1994, Kitano et al. [13] first reported laparoscopic gastrectomy for GC, and since then laparoscopic surgery has been gradually and widely used for the treatment of GC because of its advantages such as minimally invasive, less surgical-related complications, quicker postoperative recovery, and shorter hospitalization time. Following the first performance of RG by Hashizume et al. in 2003, the development of robotic equipment and the accumulation of surgical experience have made RG an increasingly popular approach in GC, which is now a large-scale surgical procedure. Currently, more and more studies have been to investigate the safety and efficacy of RG in GC treatment [14]. In the latest study, the robotic platforms engaged popularity among the digestive tract cancer surgery, especially when the tumor was located in deep pelvis, or in patients with confined pelvis. The robotic tools provide significant improved oncological and functional outcomes in rectal cancer patients, particularly in male patients [15–17].

Current research on RG suggests that it is as safe as laparoscopic surgery, has similar oncologic outcomes, and continues to have the same advantages of minimally invasive techniques. Caitlin Takahashi et al. demonstrated the advantages of RG over laparoscopic gastrectomy (LG). Advantages include lower rate of conversion to open, improved oncologic resection, and improved survival. They also demonstrated higher rates of R0 resection with the utilization of robotics in GC surgery which has a beneficial effect on patient outcomes [18]. Two randomized controlled trials in Asia, the CLASSIC and ARTIST trials, have shown that D2 lymphadenectomy performed in conjunction with adjuvant therapy improved overall survival [19, 20]. The benefit of RG includes improved instrument articulation and surgical precision which can improve overall lymph node dissection rates. The first randomized controlled trial comparing RG with LG was recently published in the Annals of Surgery in 2021 (FUGES-011), which also suggested a decrease in perioperative morbidity in RG [21]. A prospective clinical phase II study by Terashima et al. [22] demonstrated significantly lower overall complication rates and improved disease-free survival in the robotic group. This suggests that the improved oncologic resection with RG may contribute to longer recurrence-free survival.

Major surgical treatments for distal gastric cancer include Billroth-I, Billroth-II, and Roux-en-Y. The Billroth-I reconstruction has been commonly performed because of its technical simplicity, with only one anastomotic site and the ability to maintain physiological intestinal continuity [23]. Digestive tract reconstruction in totally laparoscopic total gastrectomy can be divided into two types: instrument anastomosis, which mainly employs a circular anastomosis technique and linear cutting, and hand-sewn anastomosis. Currently, instrument anastomosis is the dominant procedure, which provides convenience for surgeons, but it cannot avoid the problems associated with anvil implantation, uncertainty of the cutting edge, and high cost in special circumstances. However, in comparison to instrument anastomosis, although hand-sewn anastomosis has more requirements for the operation and a relative longer anastomosis time, it has the advantages of a good surgical field of view, can be operated in a narrow space, avoids excessive traction, and it is easy to obtain the pathology of the esophageal incisional margins before anastomosis [24].

All current researches on RG versus LG barely distinguish between reconstruction modalities, and there have been few studies on reconstruction modalities for both procedures. There are few studies on Billroth-I HA, only articles in the category of technical reports, and no comparative studies have been found so far, whereas our study is a comparative study aimed at exploring robotic versus laparoscopic access in HA.

Our study investigated the efficacy and safety of LDG-HA versus RDG-HA in Billroth-I reconstruction. The impact of baseline, pathology, perioperative data, short-term efficacy, long-term efficacy, and 5-year oncologic outcome follow-up were analyzed in both groups. There were no statistically significant differences between the LDG-HA group and the RDG-HA group in terms of clinicopathological characteristics such as age, gender, body mass index, hemoglobin, ALB, tumor diameter, previous abdominal surgery, obstruction, hemorrhage, hypertension, diabetes mellitus, smoking, alcohol consumption, ASA scores, postoperative pathology staging, degree of differentiation, vascular invasion, and neurological invasion. In our study, the time of reconstruction in the RDG-HA group was significantly faster than that in the LDG-HA group (20.80 ± 2.35 vs. 23.36 ± 4.73 min, *P* = 0.001). The main reason may be due to the flexible robotic arm of the robot made HA simpler. The total operation time was significantly longer in the RDG-HA group than in the LDG-HA group (176.55 ± 42.30 vs. 151.86 ± 31.12 min, *P* = 0.003). It mainly because that the robot has a long disassembly and loading time, and it takes a lot of time to adjust or change the instruments during the operation. While during the reconstruction of the anastomosis, the field of view was fixed, instruments were fixed, so the anastomosis time of the RDG-HA group is not affected by these factors, which takes a shorter time.

There was no statistically significant difference between the RDG-HA group and the LDG-HA group in terms of perioperative recovery and complications. It probably because that both surgical accesses were performed under the same experienced surgeon who had experiences in more than 5000 laparoscopic and 1000 robotic surgeries, which basically guaranteed homogenization of all perioperative processes in both groups except for the surgical access approach. However, the difference in average surgical duration between the two groups reached as much as 25 min. Whether this disparity would impact the incidence of complications requires further research and analysis. There were also no significant differences between the RDG-HA group and the LDG-HA group regarding OS and DFS. The reason we considered this result may be due to the fact that there was no significant difference in postoperative pathologic staging stratification between the two groups of patients.

As the first study to explore LDG-HA versus RDG-HA in Billroth-I reconstruction for GC, this study has a more single operative variable and the results were reliable. But the present study still has several limitations. First, this study was a single-center study that including only patients from our own hospital in this region and lacked validation from other medical institutions in other regions. Second, the number of patients in this study is still small, and there is still a need to continue to expand the sample size at a later stage in order to reach the goal of a multicenter study. Third, in the assessment of long-term complications, there is a potential observer bias in the assessment of gastric retention, reflux, and anastomotic stenosis due to the large subjectivity among different endoscopist. Over all, although the robotic approach shortened reconstruction time, the overall operative duration and cost remain disadvantages that require further optimization.

## Conclusion

In summary, our final results confirm that Billroth-I HA using a robotic surgical system is feasible and offers some advantages in terms of reconstruction time compared to 3D laparoscopy.

## Data Availability

All data generated or analysed during this study are included in this published article and its supplementary information files. The original data will be provided on reasonable request to corresponding author.

## Abbreviations

HA: Hand-sewn anastomosis
GC: Gastric cancer
LDG-HA: Laparoscopic distal gastrectomy hand-sewn anastomosis
RDG-HA: Robotic distal gastrectomy hand-sewn anastomosis
LDG: Laparoscopic distal gastrectomy
RDG: Robotic distal gastrectomy
OS: Overall survival
DFS: Disease-free survival
RG: Robot gastrectomy
LG: Laparoscopic gastrectomy
